# Eukaryotic 5-methylcytosine (m^5^C) RNA Methyltransferases: Mechanisms, Cellular Functions, and Links to Disease

**DOI:** 10.3390/genes10020102

**Published:** 2019-01-30

**Authors:** Katherine E. Bohnsack, Claudia Höbartner, Markus T. Bohnsack

**Affiliations:** 1 Department of Molecular Biology, University Medical Center Göttingen, Humboldtallee 23, 37073 Göttingen, Germany; 2 Institute of Organic Chemistry, University of Würzburg, Am Hubland, 97074 Würzburg, Germany; 3 Göttingen Centre for Molecular Biosciences, University of Göttingen, Göttingen, Justus-von-Liebig-Weg 11, 37077 Germany

**Keywords:** RNA methyltransferase, RNA modification, epitranscriptome, 5-methylcytosine, mitochondria, ribosome, transfer RNA (tRNA), messenger RNA (mRNA), gene expression

## Abstract

5-methylcytosine (m^5^C) is an abundant RNA modification that’s presence is reported in a wide variety of RNA species, including cytoplasmic and mitochondrial ribosomal RNAs (rRNAs) and transfer RNAs (tRNAs), as well as messenger RNAs (mRNAs), enhancer RNAs (eRNAs) and a number of non-coding RNAs. In eukaryotes, C5 methylation of RNA cytosines is catalyzed by enzymes of the NOL1/NOP2/SUN domain (NSUN) family, as well as the DNA methyltransferase homologue DNMT2. In recent years, substrate RNAs and modification target nucleotides for each of these methyltransferases have been identified, and structural and biochemical analyses have provided the first insights into how each of these enzymes achieves target specificity. Functional characterizations of these proteins and the modifications they install have revealed important roles in diverse aspects of both mitochondrial and nuclear gene expression. Importantly, this knowledge has enabled a better understanding of the molecular basis of a number of diseases caused by mutations in the genes encoding m^5^C methyltransferases or changes in the expression level of these enzymes.

## 1. Introduction

Chemical modification of nucleic acids is a key cellular process that occurs in all three domains of life. The spectrum of different modifications detected in DNA is relatively limited (six), while the range of modifications present in RNA is much higher, with more than 140 types of modification reported so far [1]. In eukaryotes, 5-methylcytosine in DNA (5mC) and its oxidized derivatives (5-hydroxymethylcytosine (5hmC), 5-formylcytosine (5fC), and 5-carboxylcytosine (5caC)) are the most prominent modifications and have been suggested to contribute to epigenetic gene regulation through a variety of different mechanisms (reviewed in [2]). However, 5-methylcytosine is also present in diverse RNA species (m^5^C; Table 1; reviewed in [3]), where it has emerged as an important regulator of many aspects of gene expression, including RNA export, ribosome assembly, translation, and RNA stability. The development of a number of 5mC/m^5^C mapping approaches, such as bisulfite sequencing, anti-m^5^C-crosslinking, and immunoprecipitation (CLIP), Aza-IP, and methylated iCLIP (miCLIP) has enabled the positions of many such modified nucleotides to be precisely defined in both the genome and transcriptome. The enzymes responsible for installation of 5mC in DNA and the functions of these epigenetic marks have been described in several recent reviews (see for example [4]). Here we discuss the current knowledge on the human RNA m^5^C modification machinery, focusing on the mechanisms of action of m^5^C methyltransferases, the cellular functions of these enzymes, and the modifications they install, as well as the implications of defects in such enzymes in disease.

## 2. Eukaryotic m^5^C RNA Methyltransferases and Their Catalytic Mechanisms

**Figure 1:**
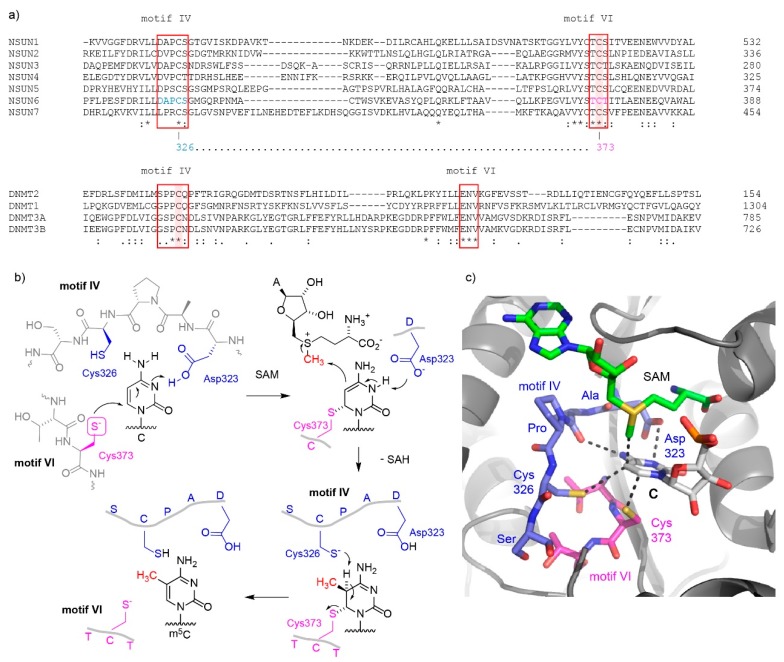
Mechanisms of C5-methylation of cytosine by m^5^C RNA methyltransferases. (**a**) Amino acid sequence alignment of regions forming the active sites of human m^5^C methyltransferases. Top: NSUN family of m^5^C RNA methyltransferases, bottom DNMT family containing DNMT2 as RNA methyltransferase and DNMT1 and DNMT3A/B as DNA methyltransferases. The conserved motifs IV and VI are boxed. The catalytic cysteine that forms a covalent bond with C6 of the target cytosine is marked with magenta background, and is located in motif VI in NSUN methyltransferases, and in motif IV in DNMT methyltransferases. (**b**) The catalytic mechanism is depicted in detail for NSUN6 (see text for description). (**c**) The active site in the crystal structure of NSUN6 with target RNA is presented, showing the arrangement and key contacts between the amino acids in the active site and the target cytosine (C72) in RNA (PDB 5WWS).

It is known that m^5^Cs in RNAs are introduced by members of the NOL1/NOP2/SUN domain (NSUN) family of proteins, which contains seven members (NSUN1-7) in humans [26], as well as the DNA methyltransferase (DNMT) homologue DNMT2. While NSUN1, NSUN2, and NSUN5 are conserved throughout eukaryotes (in *Saccharomyces cerevisiae*, named Nop2, Trm4, and Rcm1, respectively), the remaining NSUN proteins are only present in higher eukaryotes. The NSUN proteins are (putative) S-adenosylmethionine (SAM)-dependent methyltransferases that are typified by an RNA-recognition motif (RRM) and Rossman-fold catalytic core that accommodates the SAM cofactor. Mechanistically, it is proposed that NSUN proteins use two catalytic cysteines in the active site, whereas DNMT2 acts more like DNA methyltransferases that use a single active site cysteine [27,28]. In both mechanisms, a covalent intermediate is formed between a cysteine of the protein and the cytosine in RNA, in order to activate the electron-deficient pyrimidine heterocycle for the nucleophilic attack of carbon 5 on the methyl group of SAM. Interestingly, the nucleophilic cysteine that forms the covalent intermediate with the nucleoside is located in different conserved protein motifs in NSUN and DNMT proteins (Figure 1a; conserved motifs IV and VI are marked with a red box, the nucleophilic cysteine is highlighted with a magenta background).

The NSUN family enzymes use the cysteine located in amino acid motif VI for the nucleophilic attack on carbon 6 of the target cytosine in RNA [27]. In all seven human NSUN variants, the catalytic cysteine is preceded by threonine. Hydrogen bonding with the backbone carbonyl of proline and the aspartate sidechain in motif IV orients the base in the active site and assists bond formation by transient protonation of the endocyclic N3 of cytidine (Figure 1b,c; residue numbers shown for NSUN6 based on the crystal structure [21]). The activated nucleobase then accepts a methyl group from the properly positioned SAM cofactor, resulting in the formation of a carbon-carbon bond and generation of S-adenosylhomocysteine (SAH). To complete the reaction, the covalently bound methylated RNA has to be released from the protein. This elimination is assisted by the cysteine located in motif IV of NSUN proteins. This cysteine is located next to a partially conserved proline and acts as a base to deprotonate the tetrahedral carbon and initiate the elimination reaction that restores the unsaturated m^5^C heterocycle. The catalytic mechanism is supported by extensive mutational analyses. For a yeast orthologue of the NSUN1 protein, Nop2, it has been shown that the cysteine in motif VI next to threonine is essential for function [29]. Additionally, it was shown for Nop2 as well as for human NSUN2 and NSUN3 that mutation of the cysteine in motif IV to alanine or serine resulted in a stable covalent intermediate [7,12,30,31]. 

In contrast to the NSUN proteins, methyltransferases of the DNMT family do not contain a cysteine in motif VI and instead use the cysteine in motif IV as the nucleophile for attack at carbon 6. A conserved glutamate in motif VI takes the role of aspartate in motif IV of NSUN enzymes to facilitate the covalent bond formation by protonation of N3 [32]. Thus, the roles of motifs IV and VI seem to be switched in NSUN and DNMT methyltransferase families. The covalent intermediate containing the 5,6-dihydropyrimidine was characterized by structural studies using mechanism-based inhibitors, such as 5-fluoropyrimidine substrate analogs, which form a stable complex with the enzyme [33,34]. Alternatively, 5-azacytosine was used as suicide inhibitor that leads to a stable covalent crosslink between the nucleic acid and the enzyme [15,35].

## 3. Cellular Functions of m^5^C RNA Methyltransferases and the Modifications They Install

### 3.1. NSUN1 and NSUN5 Modify Cytoplasmic Ribosomal RNAs

**Figure 2:**
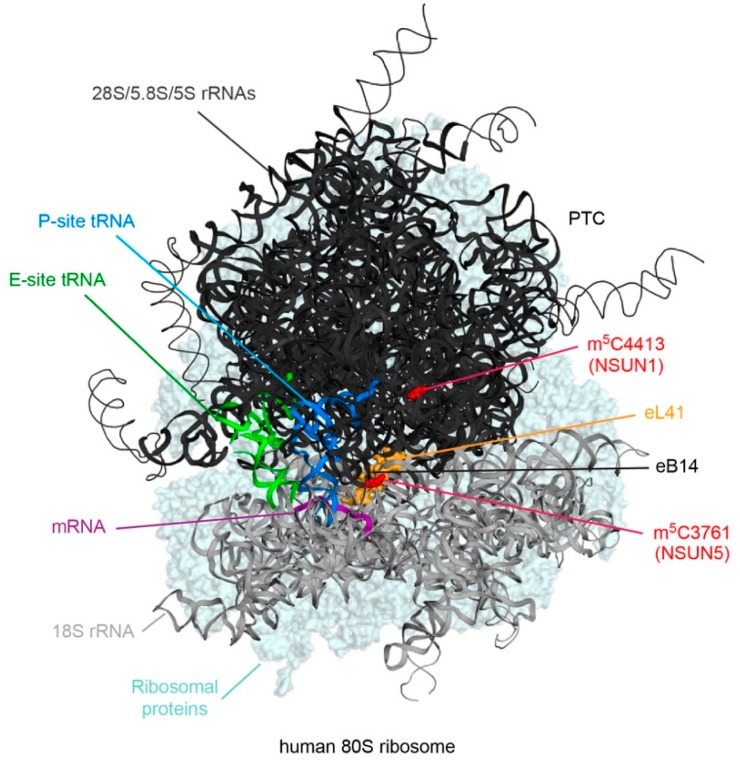
Schematic views of the positions of m^5^C modifications in cytoplasmic rRNAs. A cryo-EM structure of the human 80S ribosome is shown with the positions of 28S-m^5^C3761 and 28S-m^5^C4413, the enzymes that install them as well as key ribosomal features indicated. The ribosomal protein eL41 is highlighted in yellow, tRNAs in the P-site and E-site in blue and green, respectively, and an mRNA fragment in magenta. PTC – peptidyl transferase center; DS – decoding site; eB – eukaryotic inter-subunit bridge.

Eukaryotic cytoplasmic ribosomes are large ribonucleoprotein complexes, which are responsible for the production of all cellular proteins, and are composed of four ribosomal RNAs (rRNAs) and approximately 80 ribosomal proteins. During their maturation, the rRNAs are decorated with a cornucopia of chemical modifications, the majority of which are 2’-*O*-ribose methylations or pseudouridines, introduced by small nucleolar RNPs (snoRNPs) [36,37]. The eukaryotic rRNAs also contain a number of base modifications, including two m^5^Cs at positions 3761 (human)/2870 (yeast) and 4413 (human)/2278 (yeast) of the 28S/25S rRNA (Table 1). C5 of 28S-C3761/25S-C2278 is methylated by NSUN5 (human)/Rcm1 (yeast), whereas NSUN1 (human)/Nop2 (yeast) targets 28S-C4413/25S-C2870 [5,18,19,20]. Within the mature ribosome, these modifications lie in close proximity to the peptidyltransferase center (PTC; 28S-C4413/25S-C2870) within the large ribosomal subunit (LSU) and at the inter-subunit bridge eB14 (28S-C3761/25S-C2278; Figure 2).

On a molecular level, m^5^C stabilizes RNA structures by promoting base stacking and by increasing the thermal stability of hydrogen bonding with guanine [38,39]. It is likely, therefore, that the m^5^Cs present in the rRNAs serve to help stabilize rRNA folding within these functionally important regions of the ribosome. Consistent with this, in yeast, loss of Rcm1 influences the structural conformation of helix 69/70 of the 25S rRNA in oxidative stress conditions [18], and the combined loss of both 25S-m^5^C2278 and 2’-*O*-methylation of the nearby 25S-G2288 dramatically destabilizes the pre-LSU indicated by failure to recruit many LSU ribosomal proteins [19]. The m^5^C modification installed by Rcm1/NSUN5 is further suggested to influence ribosome function, as reporter assays have revealed that deletion of Rcm1 from yeast promotes read-through of premature termination codons [18]. Within mature yeast ribosomes, m^5^C2278 is directly contacted by the ribosomal protein eL41 (RPL41), which acts as a pivot for small subunit (SSU) rotation during translation [40], perhaps providing a mechanistic basis for how the modification influences translation. Substoichiometric modification of rRNA nucleotides is suggested to be an important source of ribosome heterogeneity. Interestingly, although quantitative mass spectrometric analysis of rRNA modification in yeast demonstrated that in vivo, 25S-C2278 and 25S-C2870 are typically 100 and >95% methylated, respectively [41], lack of Rcm1-mediated 25S-m^5^C2278 promotes the recruitment of a specific subset of mRNAs coding for proteins involved in the oxidative stress response to the ribosome. This may suggest that 25S-m^5^C2278/28S-m^5^C3761 contributes to the regulation of cytoplasmic translation and is in line with the observation that Rcm1/NSUN5 contributes to stress resistance and longevity in several model organisms [18]. Less is known about the precise function(s) of the rRNA 28S-m^5^C4413/25S-m^5^C2870 modification. Although NSUN1/Nop2 is known to be required for biogenesis of the LSU [42,43], several rRNA modification enzymes are suggested to have additional functions beyond catalyzing their target modifications [44,45], and it remains unclear whether the presence of NSUN1 or 28S-m^5^C3761 is important for LSU assembly.

### 3.2. Cytoplasmic Transfer RNAs are Methylated by NSUN2, NSUN6 and DNMT2

**Figure 3:**
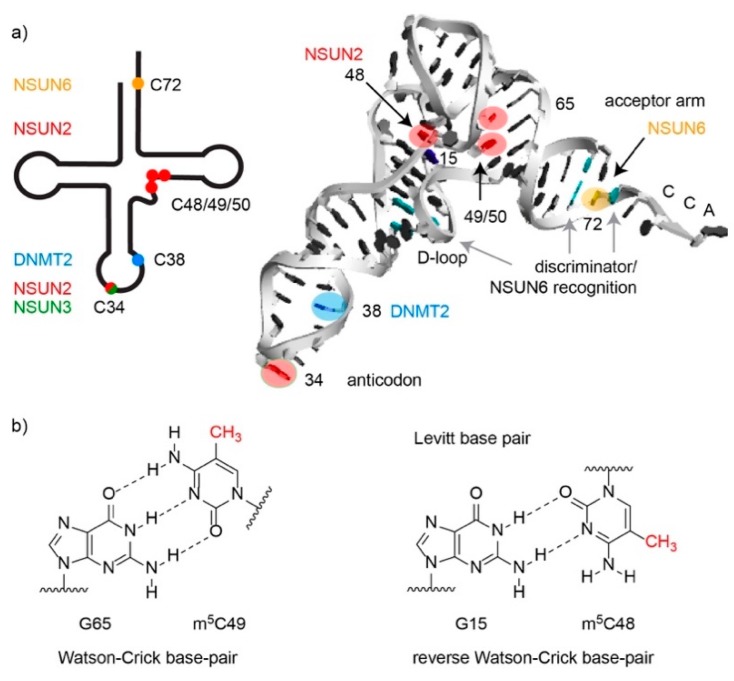
The m^5^C modifications in cytoplasmic and mitochondrial tRNAs. (**a**) Schematic secondary structure and three-dimensional L-shape structure of a tRNA with the positions of m^5^C modifications and the cognate methyltransferases responsible for installing them marked. The interaction sites of NSUN6 with the discriminator base and additional base pairs in the acceptor stem and the D-loop are indicated as observed by X-ray crystallography. (**b**) Chemical structures of m^5^C-containing base pairs in Watson-Crick orientation (with the D-stem G65-m^5^C49) and the reverse-Watson-Crick orientation of the Levitt base pair G15:m^5^C48.

Transfer RNAs (tRNAs) are the most extensively modified cellular RNAs, and three m^5^C methyltransferases, NSUN2, NSUN6, and DNMT2, have been shown to act on cytoplasmic tRNAs. While NSUN6 and DNMT2 specifically methylate C72 and C38 of particular tRNAs respectively [22,24], NSUN2 has a much broader target spectrum and is able to modify several positions (C34, C40, C48, C49, and C50) in a number of different tRNAs [6,7,8], as well as other RNA substrates (see below; Table 1 and Figure 3a). 

In the nucleus, pre-tRNAs are processed to remove 5’ leader, 3’ trailer, and intron sequences, and three non-templated nucleotides, CCA, are added to the 3’ end, which is a pre-requisite for aminoacylation. Although modification of tRNAs occurs at different stages of tRNA biogenesis, the majority of tRNA modification enzymes are nuclear, suggesting that most modifications occur during the early stages of tRNA biogenesis. In line with this, NSUN2 localizes predominantly in the nucleus, and NSUN2-mediated methylation of C34 of tRNA^Leu(CAA)^ has been shown to occur exclusively on intron-containing tRNA precursors [6]. Notably, in humans and *Drosophila melonagaster*, DNMT2 is present in both the nucleus and cytoplasm [24,46], suggesting that installation of m^5^C38 modifications could also occur during the later stages of tRNA biogenesis. Notably, NSUN6 localizes to the cytoplasm and appears enriched in proximity to the golgi aparatus and pericentriolar matrix [22], indicating that methylation of C72 residues is a late maturation event that takes place after nuclear export.

**Figure 4:**
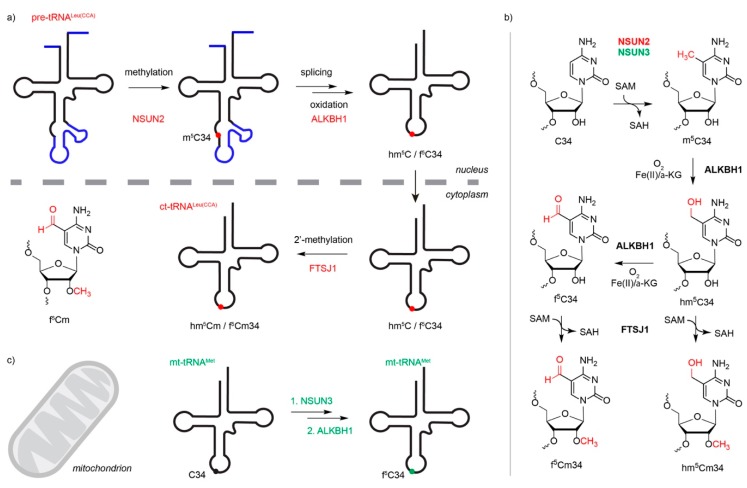
Hypermodification of m^5^C34 in anticodon of cytoplasmic and mitochondrial tRNAs. (**a**) During the maturation of the cytoplasmic tRNA^Leu(CAA)^, an m^5^C34 modification is installed by NSUN2. The methylated RNA is spliced, followed by oxidation to hm^5^C and f^5^C, which occurs in the nucleus. The oxidation is catalyzed by ALKBH1. The RNA is exported to the cytoplasm, where the methyltransferase FTSJ1 installs an additional methyl group on the ribose 2’-OH to produce hm^5^Cm and f^5^Cm. (**b**) Structures of modified nucleotides at position 34 and the modification pathway. (c) Modification of mt-tRNA^Met^ by NSUN3 and ALKBH1 occurs in the mitochondria. SAM = *S*-adenosylmethionine, SAH = *S*-adenosylhomocysteine, α-KG = alpha-ketoglutarate.

In general, modifications that lie within the tRNA core are suggested to either influence tRNA structure, stability, or both, while modifications within, or close to, the anticodon, instead contribute to tRNA function by affecting codon-anticodon interactions; tRNA^Leu(CCA)^ is the only cytoplasmic tRNA that is modified by an m^5^C methyltransferase within the anticodon [6]. However, tRNA^Leu(CAA)^-m^5^C34 is an intermediate in the formation of a hypermodification at this position; following intron-removal, the m^5^C is oxidized by the α-ketogluterate- and Fe^2+^-dependent dioxygenase ALKBH1 to produce 5-hydroxymethylcytosine (hm^5^C), 5-formylcytosine (f^5^C), or both at this position, and then after export of the tRNA to the cytoplasm, 2’-*O* ribose methylation by FTSJ1 takes place to generate 5-hydroxymethyl-2’-*O*-methylcytidine (hm^5^Cm), 5-formyl-2’-*O*-methylcytidine (f^5^Cm), or both (Figure 4a,b, [47,48]). Interestingly, wobble base modification of tRNA^Leu(CCA)^ is implicated in regulating translation [49]. In yeast, C34 of tRNA^Leu(CCA)^ is substoichiometrically modified under normal conditions, but the extent of methylation is increased upon exposure to oxidative stress. This leads to enhanced translation of mRNAs enriched in UUG codons, such as the ribosomal protein eL22a (Rpl22a), which is required for the oxidative stress response [49]. Methylation of position C38 within the anticodon loop of tRNA^Asp(GUC)^ by DNMT2 also promotes translation of a specific subset of genes, but in this case, the modification promotes association of the aspartyl-tRNA synthetase leading to more efficient aminoacylation and enhanced translation of poly-Asp-containing proteins [50]. DNMT2-mediated C38 modification has also been suggested to affect translation accuracy by facilitating discrimination between cognate and near-cognate codons; lack of tRNA^Asp^-m^5^C38 decreases the ability of tRNA^Asp^ to compete with near cognate tRNAs (e.g., tRNA^Glu^), leading to greater amino acid mis-incorporation rates [51]. Interestingly, in various species, substitution of G34 of tRNA^Asp(GUC)^ for queosine (Q) strongly increases Dnmt2-mediated m^5^C38 modification, indicating cross-talk between these two modifications [52,53] The functional significance of this interdependence is not yet fully understood, but as eukaryotes are not able to synthesize queuine and rather salvage it from their environment, the cross-talk between these modifications could suggest a mechanism by which translation regulation can be coupled with nutritional status [53,54]. Interestingly, DNMT2 re-localizes to stress granules following heat-shock and lack of m^5^C38 modification leads to increased production of tRNA fragments, suggesting that DNMT2-mediated tRNA modification plays a role in the cellular stress response [25].

All other m^5^C modifications in cytoplasmic tRNAs are present outside the anticodon loop (Table 1 and Figure 3a), and are therefore likely to primarily influence tRNA structure and stability. The m^5^C-48/49/50 modifications installed by NSUN2 cluster within the variable loop at the junction with the T-stem. A “Levitt pair” interaction between C48 and G15 in the D-loop is critical for formation of the characteristic L-shaped tertiary fold of most tRNAs [55], and it is suggested that the presence of m^5^C at position 48 increases the hydrophobicity of the base, increasing base stacking, and thereby helping stabilize this interaction and the tRNA tertiary fold (Figure 3b) [56]. Notably, NSUN2-mediated methylations within the variable loop have also been shown to protect tRNAs against stress-induced, angiogenin-mediated endonucleolytic cleavage. tRNAs lacking m^5^C48/49/50 modifications are bound more tightly by angiogenin, leading to accumulation of 5’ tRNA-derived small RNA fragments, which trigger cellular stress and are implicated in disease (see below) [7]. In contrast to the NSUN2-mediated modifications, the m^5^C72 modifications installed by NSUN6 lie within the acceptor stem, and currently, the precise role of these modifications remains elusive. Given the close proximity of C72 to the 3’ end of the tRNA and the recognition of specific nucleotides within the acceptor stem by aminoacyl-tRNA synthetases, it is tempting to speculate that NSUN6-mediated methylation of C72 may influence tRNA charging, but this was recently found not to be the case for the *Pyrococcus horikoshii* (*Ph*)NSUN6 homologue [57]. Instead, m^5^C72 was reported to promote the thermal stability of *Ph*tRNAs [57].

### 3.3. NSUN3 and NSUN4 Install m^5^Cs in Mitochondrial RNAs

Mitochondrial gene expression is essential for the production of components of the oxidative phosphorylation system. While the majority of mitochondrial messenger RNA (mt-mRNAs) coding for these proteins, as well as the 12S and 16S mt-rRNA and 22 mt-tRNAs, are transcribed from the mitochondrial genome, assembly of the mitochondrial translation machinery requires numerous nuclear-encoded proteins. Two of the seven NSUN proteins (NSUN3 and NSUN4) are synthesized on cytoplasmic ribosomes but localize to mitochondria. In the case of NSUN4, a 25 amino acid mitochondrial leader peptide that is cleaved after mitochondrial import has been identified, suggesting that NSUN4 is imported via the TOM-TIM23 pathway [58,59]. While NSUN3 has been shown to localize to the mitochondrial matrix [15], its mitochondrial import has not been investigated.

NSUN4 is responsible for installing an m^5^C modification at position 911 of the mouse 12S rRNA (equivalent to 12S-m^5^C841 in humans) [17], which lies within the decoding site of the small mitochondrial ribosomal subunit (mt-SSU). NSUN4, which in contrast to other NSUN proteins lacks an RNA recognition motif, forms a heterodimeric complex with the mitochondrial transcription factor MTERF4 [58]. MTERF4 is not, however, required for C5 methylation of mt-12S-C911 by NSUN4, suggesting that this is an independent function of the methyltransferase. Instead, MTERF4 is responsible for recruitment of the methyltransferase to the late pre-mt-LSU complexes, where the complex is required for assembly of the mature SSU and LSU into monosomes. It is not yet clear how NSUN4-MTERF4 regulate subunit joining, but this function does not require the catalytic activity of NSUN4 and it is suggested that either the heterodimer physically blocks subunit interaction or that the binding of NSUN4-MTERF4 to a specific site in the mt-16S rRNA influences activation of the mt-LSU [17]. It is possible that the dual functionality of NSUN4 in 12S rRNA methylation and mt-LSU biogenesis acts as a quality control mechanism to ensure that only fully mature mt-SSU and mt-LSU can be assembled into functional mitochondrial ribosomes. Such a model would suggest an important role for mt-12S-m^5^C911 in mitoribosome function, but while lack of NSUN4 impairs mitochondrial translation [17], this may reflect the lack of monosome production and the precise molecular role of the m^5^C modification currently remains elusive.

In contrast to NSUN4, NSUN3 is a mitochondrial tRNA m^5^C methyltransferase that specifically targets the wobble position (C34) of mt-tRNA^Met^ [14,15,16,60]. Mt-tRNA^Met^-C34 is almost fully modified in vivo, and interestingly, although bisulfite and reduced bisulfite sequencing analyses indicate the presence of some m^5^C at this position, the majority undergoes further oxidation by ALKBH1 to generate f^5^C (Figure 4c) [15,48]. In contrast to the cytoplasmic translation machinery, where two alternative tRNAs mediate incorporation of methionine either during translation initiation or elongation, due to evolutionary reduction of the mitochondrial genome, mitochondria contain only a single methionine tRNA. Furthermore, mitochondria employ a specialized genetic code, in which mt-tRNA^Met^ is required to not only recognize conventional AUG codons, but additionally is employed for decoding AUA codons during translation initiation and elongation, as well as the AUU initiation codon on the ND2 mRNA. The wobble base modification(s) installed by NSUN3 and ALKBH1 likely serve to expand codon recognition by mt-tRNA^Met^, enabling it to fulfil these diverse functions. Structural studies indicate that the presence of the formyl group may help stabilize non-conventional base pairing of f^5^C34 with the adenosine in the third position of the AUA codon [61,62]. Consistent with this, lack of NSUN3 (or ALKBH1) impairs mitochondrial translation, leading to decreased cell proliferation [14,15,16].

### 3.4. m^5^C Marks in Messenger RNAs

Alongside the long-known cap-proximal 2’-*O*-methylations, a number of other modifications, such as *N*^6^-methyladenosine (m^6^A), pseudouridine (Ψ) and *N*^1^-methyladenosine (m^1^A), have recently been detected in messenger RNAs (mRNAs) [63,64,65]. These modifications are implicated in regulating diverse aspects of the mRNA life cycle, including pre-mRNA splicing, mRNA export, translation, and mRNA stability. Although the presence of m^5^C in eukaryotic mRNAs was first reported almost 50 years ago [66], the recent development of m^5^C mapping techniques has prompted more extensive analysis. In a seminal study performed using bisulfite sequencing of RNAs derived from HeLa cells, more than 10,000 m^5^C sites in approximately 8500 mRNAs were reported [67]. Subsequently, m^5^C detection approaches have been applied to RNAs from diverse organisms and cell types, including mouse embryonic stem cells (ESC) [68,69], various mouse tissues (small intestine, heart, muscle, brain, kidney, and liver) [10,68], plants (*Arabidopsis thaliana*) [70], yeast (*Saccaromyces cerevisiae*) [71], and archaebacteria (*Sulfolobus solfataricus*) [71]. Collectively, these studies support the presence of m^5^C in mRNA, and suggest that m^5^C sites are enriched in 5’ and 3’ untranslated regions (UTRs) and are especially prominent in proximity to the translation start codon. However, the number and positions of m^5^Cs detected in these studies vary considerably, and it has also been suggested that mRNAs carry either no, or very few, m^5^Cs. While the presence of cell type-specific modifications may partly explain this variation, it is also likely that some of the observed differences arise due to limitations and biases of the currently available m^5^C mapping approaches. The extent of m^5^C in mRNA is therefore still controversially discussed, and further work will be required to resolve these issues.

The methyltransferase(s) responsible for installing potential m^5^C modifications in mRNAs have not yet been confirmed and the possible functions of m^5^Cs in mRNAs largely remain elusive. Several lines of evidence link NSUN2 to mRNA methylation. On a global level, depletion, overexpression, or expression of catalytically inactive forms of NSUN2, but not NSUN1, NSUN5, or NSUN6, was reported to alter the total amount of m^5^C detected in the mRNA pool [10]. Through in vitro methylation assays and reporter assays, NSUN2 has also been suggested to install modifications in specific mRNAs (e.g., p27 (KIPI), CDK1, p21, SHC, ICAM, p53, E2F3 and ErbB2) and the presence of these modifications was proposed to influence mRNA translation [11,12,13], however, evidence supporting the presence of these modification in endogenous mRNAs is lacking. Interestingly, it has been suggested that m^5^C modifications in mRNAs may exert their effects by influencing RNA-protein interactions. Consistent with this, the nuclear export factor ALYREF was recently show to preferentially bind m^5^C-containing RNAs, and depletion of this m^5^C “reader” protein causes nuclear retention of m^5^C methylated transcripts [10]. 

### 3.5. Modification of Other RNA Species by m^5^C Methyltransferases

The transcriptome-wide nature of the available m^5^C mapping approaches has indicated the presence of this modification in diverse non-coding RNA species, including vault RNAs (vtRNAs), enhancer RNAs (eRNAs), long non-coding RNAs (lncRNAs; e.g., XIST and HOTAIR), and small cajal body-specific RNAs (scaRNAs, SCARNA2). While the presence of these modifications often requires further confirmation, and the functions and enzymes responsible for introducing these methylations largely remain unknown, in some cases, these modifications have been analyzed in detail. The vault ribonucleoprotein complex, which is implicated in multidrug resistance, nucleocytoplasmic transport, and has been suggested to act as a scaffold for essential cell signaling pathways, is composed of three proteins and three vtRNAs. While miCLIP data from cells lacking NSUN2 identified specific sites in all three vtRNAs as targets of this methyltransferase, bisulfite sequencing only confirmed the presence of m^5^C in vtRNA1.1 and vtRNA1.3 (Table 1) [9]. Interestingly, lack of m^5^C69 modification in vtRNA1.1 was shown to affect its processing into a small RNA (svRNA4). Furthermore, svRNA4 acts analogous to a microRNA and a concomitant increase in the levels of the svRNA4 target mRNAs CACNG7 and CACNG8 was observed in NSUN2^-/-^ cells [9], demonstrating the functional importance of these m^5^C marks. In contrast to NSUN2, NSUN7 has been suggested to target eRNAs, which are short, non-coding RNAs that are linked to transcription regulation. NSUN7 was reported to methylate the Pfk1, Sirt5, Idh3b, and Hmox2 eRNAs in the context of a physical association with the transcriptional co-activator PGC-1α [23]. Depletion of NSUN7 causes significant decreases in the levels of these eRNAs and their cognate mRNAs, implying that the presence of m^5^C, if confirmed, may stabilize these transcripts, thereby promoting mRNA production. The observations that NUSN7 expression and eRNA methylation are upregulated during starvation [23] suggest that the methylation activity of NSUN7 may contribute to the adaptation of gene expression during the stress response. In addition to its well characterized role as a tRNA methyltransferase, Me-RIP experiments indicated a decrease in m^5^C methylation of the non-coding RNA, 7SK, in cells lacking DNMT2, suggesting that this enzyme could also have additional RNA substrates [72].

## 4. Substrate Recognition by m^5^C RNA Methyltransferases and Regulation of Their Activity

The identification of methylation targets for each of the human m^5^C methyltransferases, together with structural information on several of these proteins, allows insights into the ways in which these methyltransferases interact with their substrates and achieve methylation specificity. The broad-spectrum methyltransferase NSUN2 has been suggested to recognize different features in its diverse substrate RNAs. The reported NSUN2-mediated m^5^C modifications in mRNAs typically lie within highly GC-rich regions [10], suggesting that the enzyme may preferentially bind such sequences. However, all the known NSUN2-mediated m^5^C modifications in vtRNAs lie within a UCG motif [9], and mutagenic analysis of the NSUN2 target pre-tRNA^Leu^ revealed a consensus sequence of C/A/U_32_-U/A_33_-m^5^C_34_-A_35_-A_36_-G_37_ [6]. Interestingly, the NSUN2-mediated m^5^C modifications in the variable loops of its numerous cytoplasmic tRNA targets lie within diverse sequence contexts [7], suggesting that in this context, NSUN2 may recognize this structural feature of its non-intron-containing tRNA substrates, rather than a specific nucleotide sequence. The recognition of RNA secondary structures by NSUN2 is further supported by the finding that disruption of the elongated anticodon stem of pre-tRNA^Leu^ impedes methylation of C34 [6]. The presence of a stable anticodon stem was similarly found to be essential for formation of m^5^C34 of mt-tRNA^Met^ by NSUN3 [15].

Recent structural and biochemical analyses revealed that NSUN6 forms extensive contacts with its substrate tRNAs. The catalytic core and RRM domain interact with nucleotides surrounding the modification target (C72) [21], implying that they contribute to target specificity. U73, which has been termed the “discriminator base”, is critical for substrate recognition by NSUN6 (Figure 3a), and a flexible base pair (A:U or U:A) at positions 2:71 as well as a rigid base pair (C:G or G:C) formed between positions 3:70 are preferred [73]. While the binding pocket of human NSUN6 specifically accommodates U73, structural differences in *Ph*NSUN6 enable the archaeal enzyme to bind tRNAs containing either U73 or G73, thereby broadening its target spectrum compared to its human homologue [57]. Importantly, binding of NSUN6 disrupts base pairing within the tRNA acceptor stem and promotes base-flipping of C71 to make the C5 atom of the C72 nucleotide, which is normally base paired with G1, accessible for methylation [21]. Interestingly, NSUN6 also has a PUA domain that binds to the D-stem region of substrate tRNAs (Figure 3a), as well as the non-genomically encoded CCA 3’ end [21]. Consistent with this binding mode, the presence of the CCA was found to be an essential pre-requisite for methylation of tRNA^Cys^ and tRNA^Thr^ by NUSN6 [21,22]. Recognition of this post-transcriptional feature by the PUA domain may help regulate the timing of C72 modification relative to other aspects of tRNA maturation, or serve as a quality control mechanism ensuring that only correctly processed tRNAs are methylated. 

Several lines of evidence suggest that DNMT2, which specifically methylates position 38 of its substrate tRNAs, recognizes the local sequence context of its modification target. The anticodon loop sequences of tRNA^Asp(GUC)^, the canonical target of DNMT2, are perfectly conserved in species that express DNMT2 homologues, while various sequence diversions are observed in species that lack DNMT2. This evolutionary conservation strongly suggests the importance of elements within the anticodon loop for recognition or methylation by DNMT2 [24]. This model is further supported by the observation that m^5^C38 in other DNMT2 substrate tRNAs (tRNA^Gly(GCC)^ and tRNA^Val(AAC)^) also lie within a 5’-CAm^5^CGCG-3’ sequence context [8,25]. Furthermore, in addition to tRNAs, the *Dictyostelium discoideum* DNMT2 homologue binds to the U2 small nuclear RNA, which contains two stem-loop structures containing cytosines in equivalent sequence contexts to C38 within the anticodon loop of tRNA^Asp^ [74]. Interestingly, mutations within the variable loop of DNMT2-substrate tRNAs were found to reduce C38 methylation, suggesting that this structural feature also contributes to enzyme binding or substrate specificity [75]. 

The fact that only single (mt)-rRNA nucleotides have been identified as NSUN1, NSUN5, and NSUN4 substrates, together with the challenges of mutagenic studies on rRNAs, means less is known about how these enzymes recognize their targets. In the case of NSUN4, preferential binding to double stranded RNA substrates was observed in vitro [17]. However, as the modifications introduced by these enzymes occur within large ribonucleoprotein complexes, it is possible that protein-protein, as well as protein-RNA interactions, contribute to their recruitment to their sites of action. Indeed, the RNA-binding protein MTERF4 is suggested to act as a cofactor for NSUN4 [58], which in contrast to the other NSUN proteins, lacks an RRM domain. Structural analysis of the NSUN4-MTERF heterodimer identified a putative RNA-binding groove that could contribute to correct positioning of the substrate RNA in the active site of NSUN4 [76,77]. 

## 5. Roles of m^5^C RNA Methyltransferases in Development and Disease 

Consistent with the important roles that m^5^C methyltransferases play in RNA metabolism, mutations in the genes encoding these enzymes have been linked to various human diseases and changes in expression levels of m^5^C methyltransferases have been observed in various cancers. Loss of function mutations in *NSUN2* underlie several neurodevelopmental disorders (reviewed in [78]). A homozygous mutation in the *NSUN2* gene that leads to the substitution of glycine 679 for arginine (p.Gly679Arg) in the protein has been detected in individuals with autosomal-recessive intellectual disability [79]. This amino acid substitution is suggested to impede NSUN2 function by preventing localization of the protein to its site of action in the nucleolus. NSUN2 has also been linked to Dubowitz syndrome, which is characterized by microcephaly, growth and mental retardation, eczema, and characteristic facial features; a homozygous mutation in the canonical splice acceptor of exon 6 leads to use of a cryptic splice donor, instability of the NSUN2 mRNA, a significant decrease in protein levels, and reduced methylation of NSUN2 target RNAs (m^5^C47/48 of tRNA^Asp(GUC)^ [80]. In mice, the accumulation of 5’ tRNA fragments caused by lack of NSUN2-mediated tRNA methylation has been found to impair neurogenesis leading to decreased production of upper-layer neurons and reduced brain development [81], perhaps suggesting a mechanistic basis for the neurodevelopmental disorders observed in humans with impaired NSUN2 function.

Mutations in *NSUN3* that lead to either aberrant splicing and frameshifting (p.Glu42Valfs*11) or the introduction of a premature stop codon (c.295C>T/p.Arg99*) have been detected in patients with a mitochondrial deficiency disorder characterized by developmental disability microcephaly, failure to thrive, recurrent increased lactate levels in plasma, muscular weakness, proximal accentuated, external ophthalmoplegia, and convergence nystagmus [14]. Furthermore, mitochondrial disease-associated point mutations with the gene encoding mt-tRNA^Met^ that lead to A37G and C39U substitutions have been shown to impede methylation of C34 by NSUN3 [15,16]. In both cases, lack of NSUN3-mediated modification impairs mitochondrial translation, leading to reduced mitochondrial function. Interestingly, lack of NSUN3 impedes the differentiation of mouse embryonic stem cells towards the neuroectoderm lineage, implying that reduced mitochondrial translation affects the normal differentiation program [82]. 

Studies in mice show that during development, NSUN7 is expressed in a broad range of tissues [83], but in adults, is predominantly present in testis cells, especially spermatocytes and haploid spermatids. Furthermore, a chemically-induced mutation that leads to conversion of glutamine 333 to a stop codon (p.Gln333*) was shown to cause reduced sperm motility leading to sterility or subfertility [84]. Likewise, point mutations in exon 4 and exon 7 of *NSUN7* that convert valine 157 to a premature stop codon (p.Val157*) and induce a serine to alanine exchange have been identified in asthenospermic men [85,86]. While NSUN7, therefore, appears to be important for male fertility, it remains unknown whether the methyltransferase activity of NSUN7 on its eRNA targets is involved or if NSUN7 has additional cellular functions that are perturbed by these mutations.

So far, no specific disease-linked mutations have been identified in the genes encoding the rRNA m^5^C methyltransferases, NSUN1, NSUN5, and NSUN4. However, abolition of *NSUN4* is embryonically lethal and a conditional *NSUN4* knockout in mouse heart tissue was found to cause cardiomyopathy [17]. *NSUN5* lies within the Williams-Beuren Syndrome critical region, an approximately 1.5 Mb deletion at chromosome 7q11.23, raising the possibility that lack of NSUN5 or the 28S-m^5^C3761 may contribute to this multisystemic disorder [87]. Furthermore, expression of the *Drosophila melanogaster* and *Caenorhabditis elegans* NSUN5 homologs is decreased in senescent cells. This reduction of NSUN5 and its cognate RNA modification is proposed to contribute to increasing organism lifespan by promoting the translation of stress-related mRNAs, thereby negating aging-associated effects [18].

## 6. Conclusions and Outlook

The identification of RNA substrates of the seven NSUN proteins and DNMT2 has propelled forward understanding of the roles of m^5^C modifications in the regulation of gene expression by revealing important roles in cytoplasmic and mitochondrial ribosome assembly and translation, as well as in regulating tRNA stability, mRNA export, and transcription. While the development of various m^5^C mapping approaches has significantly expanded the repertoire of potential m^5^C sites within the transcriptome, the true extent and precise positions of m^5^C modifications in low abundance RNA species still requires further clarification. Although quantitative analyses suggest that the m^5^C target sites in (mt-)rRNAs and (mt-)tRNAs are typically fully modified, it is likely that m^5^Cs in other RNA species are present at sub-stoichiometric levels. This highlights the potential for differential m^5^C modification to be used to regulate the fate of particular RNA species in different conditions. This concept is further supported by the identification of ALYREF as the first m^5^C “reader” protein as well as the discovery that like 5mC modifications in DNA, m^5^Cs in RNAs can be intermediates in the generation of other modifications. The oxidized derivative of m^5^C, 5-hydroxymethylcytosine (hm^5^C), has recently been identified in *D. melanogaster* RNAs, where it was suggested to promote translation of specific mRNAs involved in basic cellular processes and embryogenesis [88]. Furthermore, 5-formylcytosine (f^5^C) has a well-established role in expanding codon recognition by mt-tRNA^Met^ during mitochondrial translation [61,62], and its presence has recently been reported in yeast mRNAs [89]. It remains to be determined if, in these contexts, m^5^C merely represents a transient intermediate or if a dynamic equilibrium between m^5^C and its oxidized products has functional relevance. In the future, it will be important to understand how the action of m^5^C methyltransferases is coordinated with other m^5^C-interacting proteins or enzymes.

## Figures and Tables

**Table 1:** Overview of human m^5^C methyltransferases and their RNA targets. Abbreviations: ribosomal RNA – rRNA, transfer RNA – tRNA, mitochondrial – mt, enhancer RNA – eRNA.

Methyl- transferase	Subcellular localization	Target RNA(s)	Modification installed	Ref.
NSUN1	Nucleolus	28S rRNA	m^5^C4413	[5]
NSUN2	Nucleus/Nucleolus	Pre-tRNA^Leu(CAA)^	m^5^C34	[6]
		tRNA^Ala(AGC/CGC/UGC)/His(GUG)/Ile(AAU)/^ ^Leu(CAA/AAG/CAG/UAA/UAG)/Lys(CUU)/ Met(CAU)/Ser(AGA/CGA/GCU/UGA)/Thr(CGT/UGU)/Tyr(GUA)^	m^5^C48	[7,8]
		tRNA^Asp(GUC)/Gln(CUG/UUG)/Lys(UUU)/Phe(GAA)/^ ^Thr(AGU)/Val(AAC/CAC/UAC)^	m^5^C48, 49	[7,8]
		tRNA^Glu(CUC/UUC)/Gly(CCC/GCC/UCC)/Pro(AGG/CGG/UGG)^	m^5^C48, 49, 50	[7,8]
		vtRNA1.1	m^5^C69	[9]
		vtRNA1.2	m^5^C27, 59^1^	[9]
		vtRNA1.3	m^5^C15, 27, 59	[9]
		mRNA	various	[10,11,12,13]
NSUN3	Mitochondria	mt-tRNA^Met^	m^5^C34	[14,15,16]
NSUN4	Mitochondria	mt-12S rRNA	m^5^C841^2^	[17]
NSUN5	Nucleolus	28S rRNA	m^5^C3761	[18,19,20]
NSUN6	Cytoplasm/Golgi	tRNA^Cys/Thr^	m^5^C72	[21,22]
NSUN7	Nucleus	eRNA (Pfk1/Sirt5/Hmox2/Idh3b)	various	[23]
DNMT2	Cytoplasm/Nucleus	tRNA^Asp(GUC)/ Gly(GCC)/ Val(AAC)^	m^5^C38	[24,25]

Note: ^1^ Detected by miCLIP but not bisulfite, ^2^ by analogy to mouse.
